# Providence Asylum

**Published:** 1886-02

**Authors:** 


### Providence Asylum.

We call attention to the advertisement of the Providence Asylum for the Insane.

This institution has existed for over twenty years, and for many years was the only institution of its kind in western New York. The good it has accomplished, and the number of persons it has restored to reason, we are unable to compute. The noble Sisters in charge have preferred to labor quietly among us, and thus the good work has gone on, with our hardly knowing the magnitude of the work accomplished. The institution has been enlarged from time to time, until now it can accommodate one hundred and fifty patients. It will be again enlarged this spring, so that it will accommodate thirty more men. Since spring, extensive repairs have been going on, and the wards are now neat and comfortable, well adapted for the treatment of the private class of insane; although the poor are received, for this is the mission of the asylum. This institution, last spring, was called upon to mourn the loss of the Sister Superior, Sister Rosalind Brown. She needs no eulogy at our hands, for she has long since received that blessed message, "Well done." For some months, Sister Mary Thomas, formerly of the Orphan Asylum in this city, has been in charge. She has shown marked ability for this work, and has received from the Superior of the Order the permanent appointment.

For twenty years the medical treatment has been conducted by Dr. William Ring, of this city, who visited the institution regularly. Under his charge many have recovered, and the proportion of recoveries has been as large as at any of the similar institutions. The medical work has so increased, that it has urgently demanded the addition of another medical man. The Sister Superior has made a most admirable selection by the appointment of Dr. Floyd S. Crego, for several years physician at the State Asylum, and now a practicing physician in Buffalo. The physicians will visit the institution alternately, and any who entrust their sick to the care of the asylum, will, we are sure, find them attentive and kind, as well as qualified in this department. Every ward is under the care of the Sisters, most of whom have been engaged in asylum work for years, as the order has several institutions of this kind under their charge. The self-denying labors of the Sisters of Charity is as beneficial here as in any hospital. We wish the institution under the new regime God-speed, and bespeak for it a liberal patronage from our readers.

---

We observe that Dr. Piffard has retired from his editorial connection with the *Journal of Cutaneous and Venereal Diseases.* The Journal will be continued under the sole editorial charge of Dr. P. A. Morrow. We may remind our readers that this is the only publication in the English language devoted to Skin and Venereal Diseases, and, during the three years of its existence, it has won for itself a high reputation for scientific excellence as well as practical utility. In addition to presenting all that is new and valuable in these special departments, the colored lithographs and wood engravings with which the original articles are illustrated are worth more than the price of subscription.

Judging from the handsome appearance of the January number, which is enriched by an admirable chromo-lithograph and a number of well-executed wood cuts, and the eminently practical character of its contents, this high standard will be maintained in the future.

